# Fracture of the Patella

**Published:** 1898-09-17

**Authors:** 


### FRACTURE OF THE PATELLA.

The interest which has been aroused in all that has to do with injuries to the patella by the unfortunate accident from the results of which the Prince of Wales is suffering must be our excuse for drawing attention to a case at the Royal Free Hospital under the care of Mr. Battle, which is recorded in the *Lancet*. The case is one of especial interest in that it shows what good results may be obtained in really bad cases when, in consequence of the severity of the injury, the surgeon is driven to adopt a radical line of treatment which he might perhaps have hesitated to undertake if the condition had at the beginning been less grave. The patient was a cabman, aged 53, who, while sitting on the driving seat of his cab, was kicked on both knees by the horse, the splash-board being destroyed. On the right knee there was a transverse lacerated wound extending almost the whole width of the limb, laying open the joint. The patella was broken transversely into two main fragments, in addition to which several smaller fragments could be felt. About five hours after the accident Mr. Battle proceeded to wire the bone. An incision three inches long was made directly upwards from the middle of the horizontal wound, the flaps were reflected on each side of this incision, and the knee joint was completely exposed. The joint cavity, the bony fragments, and the surrounding parts were then thoroughly washed in a solution of perchloride of mercury. Two or three of the smaller fragments were removed, and then the two larger pieces of bone were joined together with silver wire, the ends of the wire, after having been twisted, being hammered down on to one of the fragments. The aponeurosis over the patella was then united by silk sutures and the edges of the wound were brought together, a drainage tube being inserted through a separate incision on the outer side of the joint. A dressing of double cyanide gauze and absorbent wool was applied, and the limb was fixed in plaster of Paris. On May 3rd the stitches were removed, and the plaster sprint was exchanged for a wooden back splint. On the 12th the patient was allowed to sit up; on the 19th the splint was removed; and on May 24th, when he was discharged from the hospital, *i.e.*, less than five weeks from the time of the accident, he had firm union of the patella and was able to walk. This case is a good illustration of the advantages of the completely open method in dealing with fracture of the patella. Roughly, it may be said that, except in the aged, if there is a separation of more than a finger's width between the fragments some operative method will have to be adopted if a good and firm union between them is to be secured. It then becomes a question whether subcutaneous wiring or an open operation is the best, and, as we said in commenting on the accident to the Prince of Wales at the time of its occurrence, a strong feeling has lately grown up among many surgeons in favour of the completely open operation, the idea being that, if the risks inseparable from any operation have to be run, it is best to do an operation such as the one above described, which shall give full opportunity for removing all blood and other effusion, and of making certain that the bones are actually in contact, and that no fibrous structures are lodged between the broken surfaces. The case just mentioned shows how correct this view is, and what good success may follow such an operation, even although at the time of the accident much risk had been run of septic infection of the wound.

